# Revisited and innovative perspectives of oral ulcer: from biological specificity to local treatment

**DOI:** 10.3389/fbioe.2024.1335377

**Published:** 2024-02-22

**Authors:** Ziyi Pan, Xu Zhang, Wangni Xie, Jing Cui, Yue Wang, Boya Zhang, Liuyi Du, Wenhao Zhai, Hongchen Sun, Yunfeng Li, Daowei Li

**Affiliations:** ^1^ Jilin Provincial Key Laboratory of Tooth Development and Bone Remodeling, Hospital of Stomatology, Jilin University, Changchun, China; ^2^ School of Stomatology, Jilin University, Changchun, China; ^3^ State Key Laboratory of Supramolecular Structure and Materials, College of Chemistry, Jilin University, Changchun, China; ^4^ Joint Laboratory of Opto-Functional Theranostics in Medicine and Chemistry, The First Hospital of Jilin University, Changchun, China

**Keywords:** oral ulcer, mucosa-inspired scarless healing, ulcer-related factors, local treatment, bioadhesive polymers

## Abstract

Mouth ulcers, a highly prevalent ailment affecting the oral mucosa, leading to pain and discomfort, significantly impacting the patient’s daily life. The development of innovative approaches for oral ulcer treatment is of great importance. Moreover, a deeper and more comprehensive understanding of mouth ulcers will facilitate the development of innovative therapeutic strategies. The oral environment possesses distinct traits as it serves as the gateway to the digestive and respiratory systems. The permeability of various epithelial layers can influence drug absorption. Moreover, oral mucosal injuries exhibit distinct healing patterns compared to cutaneous lesions, influenced by various inherent and extrinsic factors. Furthermore, the moist and dynamic oral environment, influenced by saliva and daily physiological functions like chewing and speaking, presents additional challenges in local therapy. Also, suitable mucosal adhesion materials are crucial to alleviate pain and promote healing process. To this end, the review comprehensively examines the anatomical and structural aspects of the oral cavity, elucidates the healing mechanisms of oral ulcers, explores the factors contributing to scar-free healing in the oral mucosa, and investigates the application of mucosal adhesive materials as drug delivery systems. This endeavor seeks to offer novel insights and perspectives for the treatment of oral ulcers.

## 1 Introduction

A mouth ulcer refers to the disruption of the oral mucosa, which can be caused by various factors such as trauma, dentures, dehydration, viral or autoimmune disorders. Recurrent aphthous ulcer (RAU), the most common form of ulcerative illness, affects approximately 25% of young adults and a higher proportion of children (Miller et al., 1980; Slebioda et al., 2014). Affected individuals typically fall within the age range of 10–30, and the ulcers tend to recur frequently throughout their lifetime (Rodu and Mattingly, 1992). The frequency and severity of recurrence typically decline with advancing age. Furthermore, gender may influence the incidence of RAU, with a slightly higher prevalence observed in women compared to men (Rogers, 1997).

This devastating condition is characterized by painful and superficial mouth ulcerations that significantly impact the daily lives of patients (Petersen et al., 2005). Furthermore, patients undergoing radiation and chemotherapy for head and neck cancer often experience mouth ulcers, which manifest as a type of oral mucositis (I et al., 2014). Radiation treatment with a dosage of 30 Gy or chemotherapy lasting 7–10 days can induce oral mucosal discomfort in patients, leading to the need for dietary changes. Additionally, radiotherapy-induced oral mucositis typically persists for approximately 2 weeks when the radiation dosage exceeds 60–70 Gy (Scully et al., 2006). Oral ulcers can significantly impair a patient’s nutritional intake, communication, daily activities, and overall quality of life due to the mouth’s vital role in essential bodily functions such as eating (chewing and swallowing), breathing, and speaking. The impact is not only unbearable for the general population but even more severe for cancer patients. Dietary challenges can compromise their physical and mental wellbeing and significantly reduce their 5-year survival rate (Lalla et al., 2008).

The etiology of oral ulcers remains unclear. There is a consensus within the academic community that the occurrence of RAU is multifactorial, involving immune, hereditary, systemic disease, infectious, environmental, and other factors. The prevailing notion centers around immunological disorders. Numerous studies highlight immune factors, particularly cellular immune response, as the primary pathogenic mechanism (Savage et al., 1985; 1988; Dagalis et al., 1987; Pedersen et al., 1992; Taylor et al., 1992; MacPhail and Greenspan, 1997).

When ulcers occur, individuals often choose to endure the illness and pain due to its self-limiting nature. However, in the contemporary pursuit of an improved quality of life, there is a growing emphasis on seeking better therapies to alleviate pain and promote recovery. Given the unclear etiology and pathogenesis origin of RAU, the treatment primarily focuses on symptomatic relief. The therapeutic objectives include (1) pain reduction; (2) accelerated ulcer healing; and (3) extended intervals between recurrences. The topical application of corticosteroids is widely regarded as the cornerstone of RAU therapy, as systemic administration of hormonal medications can result in various side effects (Burgess et al., 1990).

Over the past few decades, a variety of agents have been employed to deliver corticosteroids, including solutions, powders, ointments, polymer films, sprayers, and hydrogels (Borges et al., 2015; Ryu et al., 2020; Hu et al., 2021). However, these agents are often washed away or diluted within an hour due to the moist and dynamic nature of the oral cavity. This not only results in missing the crucial “golden time”, which occurs within 12–24 h, but also renders a low drug concentration ineffective in achieving the desired effect (Giannola et al., 2013). Hence, there is an imperative need to develop a specific dosage form for ulcer healing that offers sustained and robust adhesion in the wet and dynamic oral cavity environment. Additionally, a comprehensive comprehension of the oral cavity’s anatomy and physiology, ulcer healing mechanisms, and key factors will facilitate the development of innovative agents.

Moreover, gaining an understanding of the healing process of mouth ulcers can provide insights into managing other medical conditions. Skin wounds often display delayed healing and hyperplastic scarring, particularly in individuals with diabetes, where elevated local glycemic and inflammatory microenvironments hinder wound healing (Liang et al., 2022). Additionally, skin pathological scars, such as hypertrophic scars, can be aesthetically unpleasing, discomforting, and sometimes need surgical intervention. Unfortunately, these operations can lead to secondary damage and may result in persistent pathological scarring in a specific subset of individuals (Gibson et al., 2018). The consequences of scar-related dysfunction and deformity can lead to financial burdens for patients, as well as significant emotional distress. Exploring the characteristics of mouth ulcers may provide innovative insights to improve the quality of skin healing, given their rapid healing rate and ability to close without scarring.

## 2 Oral anatomy and function

**FIGURE 1:**
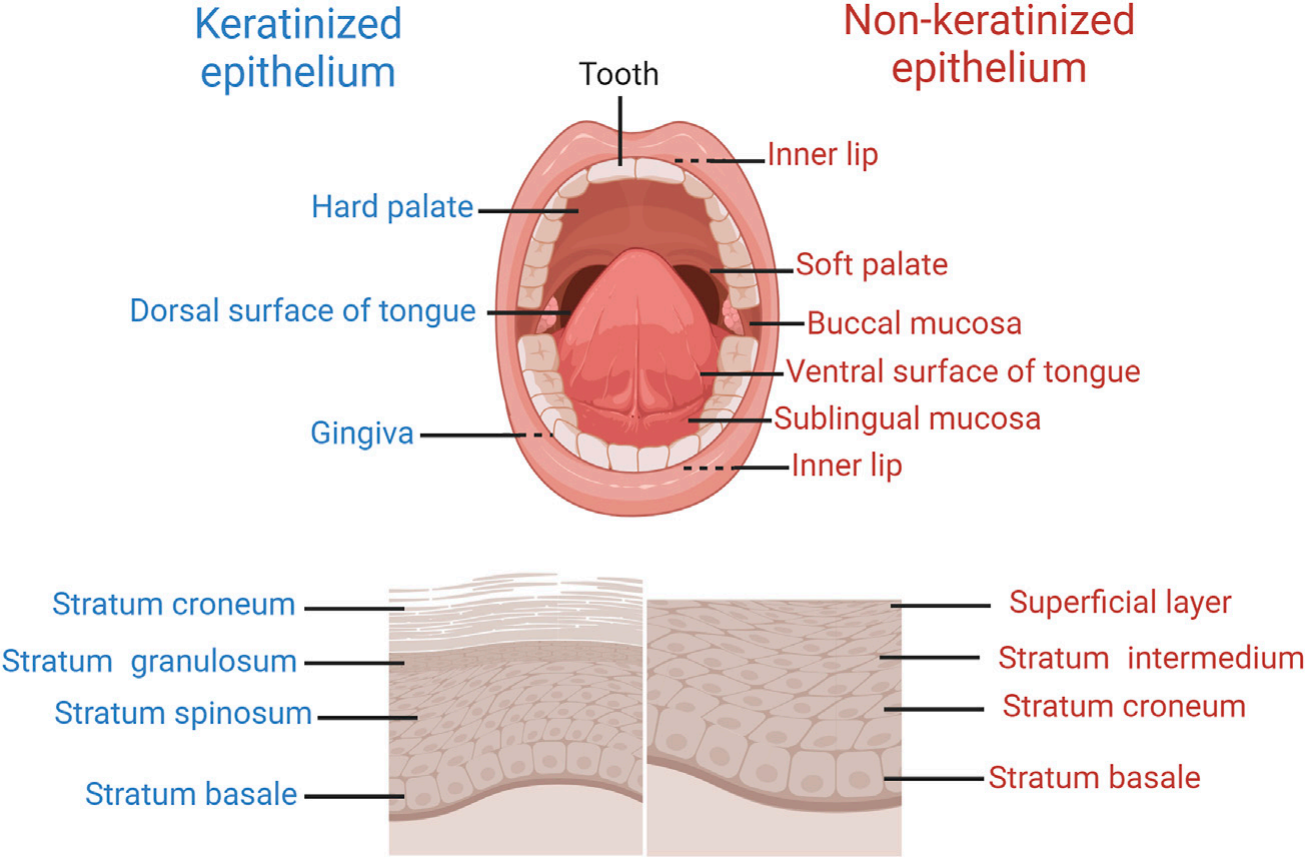
The anatomy and histology structure of oral cavity.

The oral mucosa is considered a distinctive drug delivery pathway due to its role as the primary barrier between the oral cavity and underlying tissues. The surface of the oral mucosa is covered by a layer of stratified squamous epithelium, with tightly adhering cells. The stratified squamous epithelium primarily consists of keratinocytes, along with a limited number of non-keratinocytes (Squier and Brogden, 2011). Based on histological characteristics, location, and function, it can be classified as either keratinized or non-keratinized stratified squamous epithelium. In the case of keratinized epithelium, keratinocytes are organized into four layers from deep to superficial: stratum basale, stratum spinosum, stratum granulosum, and stratum corneum. Examples of keratinized epithelium include the gingiva, hard palate, and dorsal surface of the tongue. Conversely, non-keratinized epithelium is composed of the following layers: stratum basale, stratum spinosum, stratum intermedium, and superficial layer. It covers the buccal mucosa, lips, ventral surface of tongue and sublingual mucosa (Figure 1).

### 2.1 Permeability

The epithelium of the oral mucosa consists of approximately 40–50 cell layers, and its thickness varies across different sites. The hard and soft palate mucosa, ventral tongue, and gingiva have a thickness ranging from 100 to 200 μm, whereas the buccal mucosa is thicker at 500–600 μm (Campisi et al., 2010).

Generally, tight junctions serve as the primary line of cell interaction. The oral epithelium exhibits a unique characteristic with a proliferative stratum basale. Progenitor cells, including stem cells and transiently amplifying cells, are situated in the basal layer and one to two layers above it. These cells are known as the stratum germinativum. Stem cells are present in small quantities, and upon division, their daughter cells either persist as stem cells or join the larger population of transiently amplifying cells. These cells subsequently enter the maturing population after multiple divisions and continue their migration toward the epithelial surface. Furthermore, the permeability of the epithelium can be influenced by lipid behavior observed in specific epithelial tissues, as will be elaborated later.

#### 2.1.1 Keratinized epithelium

In keratinized epithelium, cells undergo differentiation and lipid accumulation as they increase in size during migration. Within the stratum spinosum, small organelles known as membrane-coating granules or lamellar granules, which contain certain lipids, can be observed (Ricardo Martinez and Peters, 1971). At the junction between the stratum granulosum and the stratum corneum, the membrane-coated granules migrate toward the top region of the keratin-forming cell. Subsequently, the organelle membranes begin to fuse with the cytoplasmic membrane, resulting in the extrusion of lipid sheets into the extracellular space through a process referred to as cytosolic vomiting (Landmann, 1988). Upon enzymatic treatment, these lipid sheets transform into a mixture of nonpolar lipids, including ceramides, cholesterol, and fatty acids. This mixture occupies approximately half of the intercellular space between stratum corneum cells, resulting in the formation of wide lipid sheets, while the remaining half is occupied by desmosomes (Clausen et al., 1986).

#### 2.1.2 Non-keratinized epithelium

While in non-keratinized epithelium, the lipids primarily consist of polar molecules such as cholesterol esters, cholesterol, and glycosphingolipids. Moreover, the presence of membrane-coated granules is less in non-keratinized epithelium compared to the stratum spinosum of keratinized epithelium. A small number of granules contain minute lipid sheets. Upon extrusion into the extracellular space, these lipid sheets are more likely to adopt an amorphous lipid structure rather than forming distinct sheets (Wertz et al., 1993). Evidence indicates the presence of electron-lucent material in non-lamellar phase lipids within non-keratinized epithelium, characterized by sporadic short layers of lipids. This distinct and irregular lipid structure may account for the relatively higher permeability of non-keratinized epithelium when compared to keratinized mucosa. Permeability can be influenced by both the thickness of the epithelial and histological structures. The permeability of various regions is presented in the accompanying Table 1.

**TABLE 1:** Oral structure features.

Tissue	Structure	Thickness (μm)	Permeability
Buccal mucosa	Non-keratinized	500–600	+
Sublingual mucosa	Non-keratinized	100–200	++
Gingiva	keratinized	100–200	-
Palate	keratinized	100–200	-

### 2.2 Oral environment

The oral environment is characterized by two key factors: humidity and dynamics. These factors also present significant challenges for oral mucosa repair.

#### 2.2.1 Humidity

Saliva is a biological fluid produced in the mouth by various salivary glands, including the parotid gland, sublingual gland, submandibular gland, and other minor salivary glands. Saliva is continuously secreted, dispersed, and eliminated from the mouth, exhibiting a wide array of properties. Physiologically, the mouth mucosa is covered by mucus, a part of saliva that is secreted by both major and minor salivary glands. Mucus consists of approximately 95% water by weight. The mucus layer has a thickness ranging from 50 to 450 µm and is composed of water, enzymes, glycoproteins, electrolytes, and mucin (Cornick et al., 2015). The 3D network structure of mucus is formed by interconnected mucin fibers, with each fiber containing glycosylation regions and non-glycosylated hydrophobic regions. The glycosylation regions of mucin fibers are characterized by a high content of PTS (Proline, Threonine, Serine) repeat sequences in the protein backbones. The terminals of mucin fibers contain sulfate and sialic acid residues, which contribute to the negative charge of the PTS domain (Argüeso, 2010). Non-glycosylated hydrophobic regions contain numerous cysteine residues that serve as sites for disulfide bond formation. The cysteine-rich domain facilitates the separation of hydrophobic regions from one another. This results in the formation of an entangled network of mucin fibers (Argüeso, 2010).

Due to the presence of hydrophilic and hydrophobic regions, mucus can trap pathogens and various molecules through multiple interactions. Additionally, the mucus present in the oral mucosa serves as a lubricant, facilitating cellular movement between adjacent cells and protecting cell junctions from disruption. Furthermore, mucus can act as a barrier that either enhances or reduces drug absorption through various interactions. The specific outcome relies on the properties of the carriers and cargoes (Patel et al., 2011).

#### 2.2.2 Dynamic

This unique characteristic arises from two factors: saliva flow and mucosa movement. Firstly, saliva is continuously produced and flows within the oral cavity. The limited absorption efficiency comes from the rapid turnover cycle of saliva, which impacts the concentration of medicine at the absorption site. Additionally, the pH value of saliva can affect the concentration of drugs at the specific site. Moreover, the flow rate and pH of saliva vary at different time throughout the day, and food or drink consumption can lead to drug dilution (Aframian et al., 2010). On the other hand, various daily oral physiological activities like chewing, swallowing, and speaking can impact the stability of the drug-loading carrier in oral environment.

## 3 Clinical classifications

The most common ulcerative disease in clinical practice is RAU. In addition, there are other ulcerative diseases, including Behçet’s disease, traumatic ulcer, oral squamous cell carcinoma, and tuberculosis ulcer.

RAU typically manifests in three clinical subtypes: minor, major, and herpetiform (Table 2). The most common subtype is minor aphthous ulcer, which primarily occurs on non-keratinized epithelial mucosa, including the lips, tongue, buccal mucosa, and soft palate (Dreizen, 1981). Initially, the affected mucosa becomes edematous, accompanied by a burning sensation, which is followed by the development of superficial ulcers. Healing typically initiates within approximately 5 days, leading to contraction of the wound surface, redness, swelling, reduced pain, and complete healing of the ulcer within 10–14 days without scarring (Graykowski et al., 1966). Minor RAU generally does not present with significant systemic symptoms or signs. However, major RAU lesions are larger and deeper, leading to increased pain, delayed healing, and occasional scarring due to their deeper location within the affected area (Graykowski and Hooks, 1978). Herpetiform ulcers, on the other hand, are characterized by numerous shallow ulcers scattered across the affected area, resembling a starry sky (Bell and Rogers, 1982).

**TABLE 2:** RAU subtypes and clinical features.

Type	Clinical features				
	Size (mm)	Number	Duration(d)	scar	Percentage
minor	5–10	<10	10–14	×	75–85
major	>10	<10	>14	√	10–15
herpetiform	<5	>10	10–14	×	5–10

Behçet’s disease is a chronic vascular inflammatory disorder. In the oral cavity, it manifests recurrent oral ulcers. Ocular symptoms include iridocyclitis, conjunctivitis, and keratitis. Genital mucosal ulcers are also common. Skin lesions, such as nodular erythema, are frequently observed. Additionally, Behçet’s disease can affect the joints, cardiovascular system, gastrointestinal tract, and nervous system (Mendoza-Pinto et al., 2010).

Traumatic ulcer is an ulcerative disease of the oral mucosa caused by mechanical, physical, chemical, and other local irritants (Mortazavi et al., 2016). The morphology of the ulcers often conforms to the chronic mechanical injury factor, and they are surrounded by an inflammatory proliferative reaction.

In oral squamous cell carcinoma, ulcer is usually extensive and deep, and it develops rapidly. At the base, there are small, grain-like projections that resemble a cauliflower. The edges are raised, and there is a firm lump when touching the base. The nearby lymph nodes feel hard and are connected to the lesion (Bagan et al., 2010).

For oral tuberculosis ulcer, the patients usually had a history of tuberculosis. The ulcers are commonly found on the tongue and are chronic and persistent. The boundaries of the ulcer are well-defined or linear, and there is dark red granuloma visible. The edges are raised and have a “rat-bite” appearance (Eng et al., 1996).

## 4 The healing mechanism of oral ulcer

### 4.1 Stages of oral ulcer healing

**FIGURE 2:**
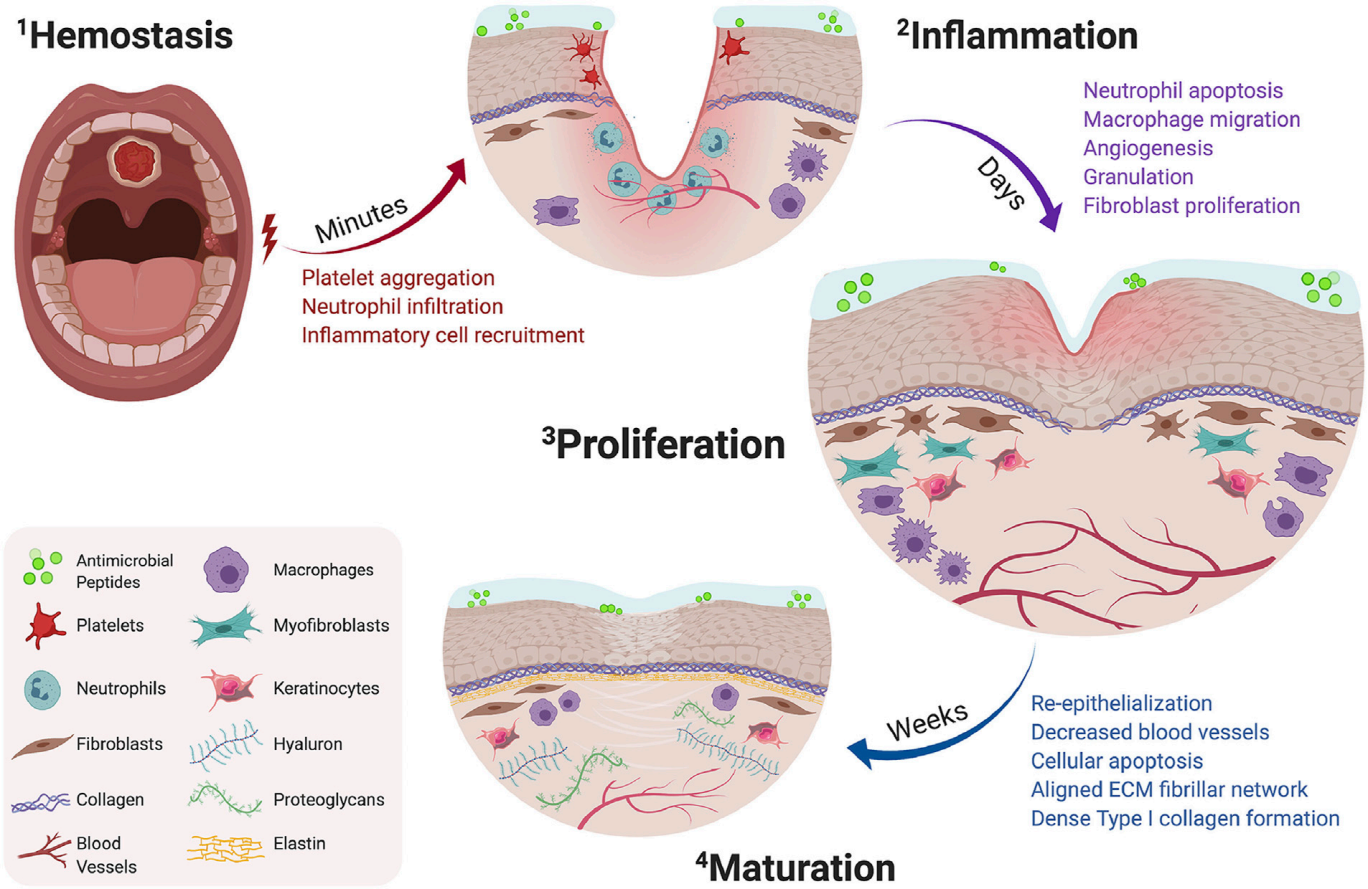
Timeline of oral wound healing and oral mucosal remodeling. Following injury, the hemostatic cascade is initiated to prevent excessive bleeding at the wound site (2-1). In the days following injury, inflammation peaks through neutrophil debridement and macrophage-mediated secretion of inflammatory cytokines (2-2). Within a week, the proliferation phase promotes fibroblast migration, increases vascular networks by angiogenesis, and enhances macrophage migration (2–3). Following fibroblast migration, the tissue surrounding the defect begins to re-epithelialize and mature by aligned fibrillar and dense collagen networks (2–4). Copyright (2021) Elsevier.

Wound healing occurs through several distinct but interconnected phases, including hemostasis, inflammation, proliferation and maturation stages (Velnar et al., 2009a) (Figure 2). While the oral mucosa undergoes stages of healing similar to the skin, it possesses unique characteristics.

#### 4.1.1 Hemostasis

Following an injury to the blood vascular endothelium, the subendothelial extracellular matrix (ECM) is exposed. The matrix components such as collagens activate the circulating platelets to initiate the hemostatic cascade and local vasoconstriction (Desjardins-Park et al., 2019). Fibrin clots ensure hemostasis as part of the hemostatic cascade and serve as the foundational matrix structure, facilitating the invasion and recruitment of inflammatory cells and other components. Concurrently, the platelets trapped within the clot continuously release bioactive products, including vasoactive mediators, proteases, cytokines, and growth factors (Broughton et al., 2006a; Goldberg and Diegelmann, 2017). Finally, the fibrin clots serve as a transient ECM scaffold, facilitating the migration of epithelial cells and fibroblasts to the wound site, while also serve as a reservoir for critical growth factors (Constantinus et al., 2016).

#### 4.1.2 Inflammation

The intensity of inflammation reaches its peak within 24–48 h after injury formation and persists for several days (de Farias Gabriel et al., 2019). Concurrently with the hemostasis stage, activated inflammatory cytokines recruit immune cells to the site of injury. These cytokines enhance blood vessel permeability, resulting in characteristic inflammatory symptoms such as redness, swelling, heat, and pain (Constantinus et al., 2016).

Neutrophils are the initial immune cells to appear, secreting numerous proteases, including matrix metalloproteinases (MMPs), which aid in the removal of microorganisms from the wound site and the breakdown of ECM components (Wilgus et al., 2013). After neutrophils, the monocytes, which typically appear after injury, undergo maturation and differentiation into macrophages. Initially, macrophages exhibit a predominantly pro-inflammatory M1 phenotype, which is helpful to innitiate inflammation and clear pathogens. In the later phase of inflammation, M1 macrophages transition their phenotype to anti-inflammatory M2 macrophages. M2 macrophages secrete abundant anti-inflammatory cytokines, including IL-10, and contribute to the downregulation of anti-inflammatory cytokine levels at the wound site (Koh and Ann, 2011). Additionally, numerous cytokines are produced by M2 macrophages, including IL-1, IL-6, fibroblast growth factor (FGF), epidermal growth factor (EGF), transforming growth factor β (TGF-β), platelet-derived growth factor (PDGF), and vascular endothelial growth factor (VEGF). They coordinate the migration of fibroblasts and endothelial precursor cells to the injury site, initiating the proliferation process (Barrientos et al., 2010). At this stage, the wound repair process shifts from the inflammatory expansion phase to the phase of inflammation resolution and tissue repair.

#### 4.1.3 Proliferation

The hallmark of this stage is the development of granulation tissue. This highly vascularized tissue replaces the previous fibrin clot. Regenerative growth factors, such as FGF, EGF, and VEGF, produced by M2 macrophages, play a role in the remodeling of granulation tissue (Desjardins-Park et al., 2019). Also, fibroblasts recruited by macrophages aid in the formation of the ECM. The newly formed ECM comprises hyaluronic acid, type I and III collagen, and fibronectin (Velnar et al., 2009b). Subsequent to migration, fibroblasts differentiate into myofibroblasts, initiating the process of wound contraction (Alexis et al., 2014). This marks the conclusion of the proliferation phase and the commencement of the maturation phase.

#### 4.1.4 Maturation

Serving as the final and longest phase of wound healing, the granulation tissue functions as a temporary scaffold, facilitating the migration of fibroblasts and other cells to the wound site and the remodeling of newborn ECM (Velnar et al., 2009c). Additionally, the granulation tissue exhibits a higher abundance of fibronectin, type III collagen, elastin, proteoglycan, and hyaluronic acid in comparison to the mature ECM. And the new granulation tissue has higher water content and lower density. Numerous proteases, including MMPs, play a role in regulating the balance between ECM degradation and deposition (Mccarty and Percival, 2013). Over time, as fibroblasts and part of macrophages undergo apoptosis, the initially disorganized deposited matrix proteins are crosslinked by fibroblasts (Broughton et al., 2006b; Xue and Jackson, 2015; Constantinus et al., 2016). Consequently, the initial temporary ECM transforms into a more extensive and denser network of type I collagen. The remaining cells, such as keratinocytes and macrophages, continue to participate in ECM remodeling (Velnar et al., 2009c; Xue and Jackson, 2015). Ultimately, with the assistance of cells and cytokines, the injury site reestablishes homeostasis.

## 5 Oral mucosa-inspired skin scarless healing

Unlike skin injuries, oral ulcers typically do not leave scars after healing. Scar tissue is characterized by disorganized collagen (predominantly type I and III), proteoglycans, and an excessive accumulation of persistent myofibroblasts, leading to abnormal tissue function (Häkkinen et al., 2000). Unlike the dermis of the head and neck, which scars due to skin injuries, injuries to the oral mucosa typically undergo faster epithelialization and rapid healing, despite the presence of constant physical trauma and bacteria. The regenerated tissue in the oral cavity is typically indistinguishable from the tissue before injury (Wong et al., 2009). Unlike cutaneous injuries, mouth ulcers exhibit distinctive genomic expression patterns that indicate rapid wound-closure and minimal scarring (Whitby and Ferguson, 1991). The timely closure of wounds has demonstrated significant benefits, including the prevention of microbial invasion, reduction of scar formation, and facilitation of wound healing (Zhao et al., 2023).

Furthermore, a well-documented disparity between healing processes in oral mucosa and skin is the comparatively reduced inflammation observed in oral mucosal wounds. This manifests as a lower presence of infiltrating inflammatory cells and reduced levels of inflammatory cytokines, including interleukin 1α and 1β (IL-1α, IL-1β), tumor necrosis factor-α (TNF-α), and chemokines such as keratinocyte chemoattractant (KC) (Chen et al., 2010a). Additionally, saliva, which is unique to the oral cavity, contains histones, antimicrobial peptides, and mucins that facilitate fibroblast proliferation and migration, promoting the renewal of keratin-forming cells and the release of growth factors (Brand et al., 2014; Bojnk et al., 2016).

**FIGURE 3:**
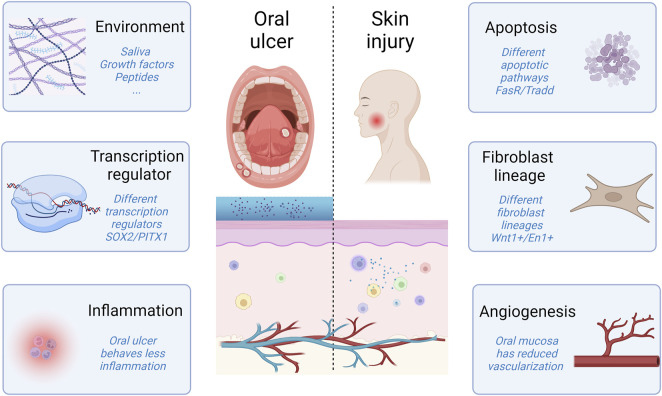
Comparison between oral ulcer and skin injury.

The unique properties of the oral cavity indicate a distinct wound healing process, where each phase focuses on rapid wound resolution. Thus, exploring the histological and environmental disparities between cutaneous and oral environments would contribute to elucidating the underlying biological mechanisms and offer novel perspectives for managing chronic and non-healing wounds (Figure 3).

### 5.1 Less inflammation

Chronic inflammation is a characteristic feature of delayed wound healing, and an overactive immune response can lead to prolonged healing, excessive fibrosis, and scar formation, all of which impede cutaneous wound healing (Eming et al., 2007). In most mammals, the reparative response post-birth does not result in tissue regeneration; instead, it often leads to scar formation accompanied by partial loss of organ function. In contrast, the reparative response in the fetus is primarily characterized by scarless regeneration due to the absence of an inflammatory response during fetal development (Colwell et al., 2003). Fetal wounds exhibit downregulation of pro-inflammatory factors such as IL-6 and IL-8, along with an increase in the anti-inflammatory factor interleukin 10 (IL-10) (Szpaderska et al., 2003). Therefore, the absence of an inflammatory condition is a prerequisite for regeneration and the absence of scarring (Koh and Ann, 2011). Transcriptomic analysis reveals the presence of multiple antimicrobial defensive immune responses in the undamaged oral mucosa, which may be attributed to immune tolerance established through the long-term maintenance of microbial homeostasis in the oral environment. In contrast, the cutaneous tissue shows low inflammatory activity in the steady state but experiences upregulated inflammation levels during the process of damage healing. This indicates that the cutaneous tissue exhibits a greater degree of chronic inflammatory response during the healing process compared to the healing of the oral mucosa. Likewise, the healing process of mouth ulcers is characterized by a mild inflammatory response, with reduced infiltration of macrophages, T cells, and neutrophils, as well as low expression of *TGF-β1* in oral epithelial cells (Chen et al., 2010a; Turabelidze et al., 2014). Additionally, skin and oral mucosal keratinocytes exhibit disparate intrinsic responsiveness to inflammatory stimuli. Stimulation of both cell types with IL-1β *in vitro* revealed significantly higher expression levels of *IL-6* and *TNF-α* in skin keratinocytes compared to oral mucosal keratinocytes. This data supports the notion that keratinocytes from the skin and oral mucosa display distinct reactivity at the site of injury through diverse regulatory pathways, leading to varying levels of inflammation depending on the site of injury (Chen et al., 2010b).

### 5.2 Different mechanisms of apoptosis

Cell death and elimination mechanisms, facilitated by paracrine signaling or immune modulation, are believed to play a crucial role in scarring outcomes. The development of fibrosis is associated with the accumulation of myofibroblasts, which contribute to the synthesis of ECM components, tissue contraction, and functional impairment. Prolonged and inappropriate resistance to apoptosis in myofibroblasts impedes their clearance and exacerbates the fibrotic response (Laplante et al., 2010). The current study suggests that oral mucosal wounds exhibit distinct apoptotic mechanisms compared to cutaneous wounds, implying that the apoptotic characteristics of specific cells may influence scar formation (Johnson et al., 2014). Comparative analysis of oral wound healing and cutaneous wound healing revealed significant alterations in the gene expression of key mediators involved in both intrinsic and extrinsic apoptotic pathways. The expression of genes involved in both intrinsic and extrinsic apoptotic pathways is frequently reduced during oral wound healing and exhibits faster restoration to baseline levels compared to cutaneous wounds. In oral wound healing, the peak expression of genes associated with intrinsic apoptosis typically coincides with the peak of inflammatory cells at 24 h. This suggests a potential relationship between the decline of inflammatory cells in the wound bed and the resolution of inflammation during the peak of intrinsic apoptosis.

Conversely, the mediators of the extrinsic apoptotic pathway were significantly upregulated in both cutaneous and oral wound healing, in contrast to the intrinsic apoptotic pathway. However, the specific mediators varied between the two types of healing. The gene expression of *Tnfrsf1b* and *Casp8* reached its peak within 24 h of both oral and cutaneous wound healing, with higher expression in the skin. Similar to the intrinsic pathway, apoptosis within 24 h is correlated with the resolution of inflammation. In skin wound healing, the expression of the *FasR* gene peaked at 24 h, whereas oral wound healing did not exhibit a corresponding peak. This phenomenon suggests that *Fas*-mediated apoptosis consistently plays a crucial role in skin wound healing. This apoptotic pattern may be associated with the apoptosis observed during vascular regression. Unlike the oral mucosa, cutaneous wounds necessitate the removal of excessive neovascularization resulting from robust angiogenesis. As skin wound healing progresses to the remodeling stage, excessive vascular regression leads to the demise of a significant proportion of endothelial cells. Additionally, a peak in the expression of *Tradd* genes was detected in oral wound healing, whereas skin wound healing did not exhibit a similar peak. In cutaneous wound healing, the *Tradd* gene may serve as a restricting factor for exogenous apoptosis, whereas in oral wound healing, its expression may be excessive. In conclusion, distinct apoptotic behaviors may correspond to specific physiological properties of various tissues during the healing process.

### 5.3 Different fibroblast lineages

Fetal wounds are characterized by the absence of thrombosis and inflammatory responses, along with a unique spatial and temporal extracellular matrix architecture and lower expression of *TGF-β*. Numerous studies have demonstrated significant differences between fetal and adult fibroblasts concerning crucial properties, including migratory activity, responsiveness to cytokines, and production of cytokines and matrix macromolecules. Research suggests that adult gingival fibroblasts in the papillary connective tissue exhibit numerous similarities to embryonic fibroblasts, such as growth and migratory characteristics, cellular shape, synthesis of migration-stimulating substances, cytokine-mediated motogenic activity, and cytokine production and response (Schor et al., 2008). Moreover, both oral mucosal and gingival fibroblasts exhibit similar contraction rates of the three-dimensional collagen matrix to dermal fibroblasts, comparable to the activity of embryonic fibroblasts. Animal wound models have demonstrated a faster rate of cell infiltration in orally derived fibroblasts compared to skin-derived fibroblasts, as observed through *in vitro* cultivation (al-Khateeb et al., 1997). Additionally, gingival and dermal fibroblasts differ in the secretion patterns of various collagenases, including matrix metalloproteinase 13, which is typically present in 7- and 14-day human gingival defect models but is absent in skin wounds (Ravanti et al., 1999). Furthermore, the inoculation of gingival fibroblasts into three-dimensional fibrin matrix results in rapid matrix restructuring and degradation, attributed to the robust expression of tissue fibrinogen activator. Conversely, matrix rearrangement and fibrinolysis occur at a slower pace in dermal fibroblast-inoculated matrices (Lorimier et al., 1996). These observations indicate that fetal and gingival fibroblasts play crucial roles in tissue healing and possess distinct characteristics compared to skin fibroblasts. Within adult tissues, gingival fibroblasts may exhibit genetic differences in specific cells, resulting in accelerated wound healing and minimal scarring.

Further investigation of surface markers and developmental genealogy has revealed that the fibroblast lineage of the mucosa, known as Wnt1 lineage positive fibroblasts (WPFs), differs from the skin fibroblasts known as En1 lineage positive fibroblasts (EPFs), which are responsible for fibrotic outcomes following injury. Reports indicated that transplantation of oral-derived fibroblasts into dorsal skin wounds resulted in minimal scarring, whereas transplantation of purified back skin-derived fibroblasts into the oral cavity led to the formation of ectopic scars (Jiang and Rinkevich, 2020). These observations suggest that different fibroblast lineages possess inherent fibrillogenic or anti-fibrillogenic activity regardless of their surrounding environment. Another study additionally demonstrated that doxycycline reduces the abundance of EPFs during wound healing, thereby reducing scar thickness (Moore et al., 2020). In doxycycline-treated wounds, the structure of collagen fibers in the extracellular matrix closely resembles the multi-branched, randomly arranged “reticular” pattern of collagen deposition observed in uninjured skin, which may account for the maintenance of scar strength despite reduced collagen content in scars. This phenomenon could be attributed to the potential collagenase-inhibitory properties of doxycycline.

### 5.4 Different environment factors

Similar to animals instinctively licking their wounds with saliva, the distinctive characteristics of the oral environment may facilitate ulcer healing. Experimental analysis revealed that the wound healing process in the oral mucosa was delayed in animal models with xerostomia or sialadenectomy (Epstein and Scully, 1992; Bodner et al., 1993). The unique environment of the oral cavity provides favorable conditions that promote healing. In the following section, we will analyze various environmental factors that potentially contribute to healing.

#### 5.4.1 Moist surroundings

Like animals instinctively licking their wounds with saliva, the unique characteristics of the oral environment have the potential to facilitate ulcer healing. Maintaining a moist wound environment offers several advantages, including preventing tissue dehydration and apoptosis, accelerating vascular rejuvenation, and enhancing the breakdown of fibrin and tissue debris. Studies have demonstrated that saliva accelerates re-epithelialization and extracellular matrix remodeling (Brand et al., 2014). Winter’s research as early as 1962 provided evidence of the beneficial impact of moist environments on wound healing (Winter 1962). Wounds exposed to moisture exhibited twice the healing rate compared to those exposed to dry conditions. In the case of wounds left exposed to air, a dry scab forms over the wound surface within 24 h. Exudation initiates in the dermis beneath the wound surface, and polymorphonuclear leukocytes in the exudate migrate upward, accumulating in the fibrous tissue. Epidermal cells at the wound margin and in the follicle traverse the fibrous tissue beneath the layer of leukocytes. Consequently, the scab comprises superficial fibrous tissue and encloses the original wound surface, resting atop the newly formed epidermis.

Conversely, in a moist environment, the epidermis migrates across the fibrous tissue of the dermis facilitated by the wound surface exudate. Leukocytes exit the epidermis and enter the exudate, resulting in the formation of minimal scabs containing fibrous tissue. Aquaporin (AQP) is an intrinsic membrane protein situated in the cell membrane. It functions by creating “pores” that regulate the movement of water into and out of the cell, essentially acting as a “cellular water pump”. Numerous studies have focused on creating a moist environment to enhance healing by upregulating the level of aquaporins. Saher et al. suggested that erythropoietin (EPO) can stimulate aquaporin3 expression, thereby promoting diabetic wound healing through accelerated cell migration, proliferation, and re-epithelialization (Hamed et al., 2017). Artificial aquaporin 3 molecules are capable of functioning on cell membranes with comparable water-selectivity and high permeability to natural AQPs, thereby aiding in wound closure (Yan et al., 2020).

#### 5.4.2 Unique components

Besides creating a warm and moist environment, saliva and its unique physicochemical properties contribute to the promotion of wound healing. Several characteristics of saliva, such as pH, ionic strength, and the presence of calcium and magnesium ions, likely play pivotal roles in this process (Edgar, 1992). Additionally, saliva reduces the redox activity induced by transition metal ions and inhibits the production of free radicals (Nagler et al., 1997). However, it has been demonstrated that even after treating the wounds separately with saliva and saline, the saliva group displayed faster wound healing rates. This finding suggests that moisture and ionic strength may not be the primary factors influencing wound healing promotion (Varshney et al., 1997). Hence, it is plausible to hypothesize that oral cavity-specific components may have a significant role to play.

##### 5.4.2.1 Growth factors

Furthermore, the pro-healing properties of saliva can be attributed to the presence of growth factors that facilitate cell division and migration. These growth factors include EGF, transforming growth factor-α (TGF-α), basic fibroblast growth factor (bFGF), PDGF, and VEGF. Enzyme-linked immunosorbent assay (ELISA) has been utilized to illustrate the disparity in cytokine release behavior between mucosa and skin. The results reveal that the oral mucosa exhibits a high rate of growth factor release (Kong et al., 2019). Furthermore, a separate study conducted at the genetic level revealed a notable upregulation of bFGF and VEGF-related genes in the oral mucosa when compared to the skin on the back. This finding suggests a higher secretion of cytokines, leading to the consideration of carefully designed therapeutic interventions by researchers (Veith et al., 2019).

##### 5.4.2.2 Peptides

Additionally, saliva contains proteins that possess anti-inflammatory properties and facilitate cell migration. These proteins include secretory leukocyte protease inhibitors, trefoil peptides, and histatins. Specifically, histatins, which are peptides with antimicrobial properties, have been shown to promote the migration of epithelial cells and fibroblasts, thereby facilitating wound closure while minimizing scarring (Oudhoff et al., 2008; Boink et al., 2016). An important characteristic that sets histatins apart from other conventional growth factors is their ability to easily form chemical variants. Transitioning from a linear to a cyclic form can enhance their biological activity by up to 1000-fold (Oudhoff et al., 2009).

Numerous researchers have conducted investigations on the impact of growth factors in promoting scar-free healing through *in vitro* experiments (Kong et al., 2019; Monavarian et al., 2019). However, there are still limitations. Applying cytokine doses established *in vitro* to treat wounds *in vivo* would result in significant errors due to the evident disparity in cellular growth states between *in vivo* and *in vitro* conditions. Moreover, the number of cytokine types investigated in these studies is insufficient to offer a comprehensive understanding of how different cytokines impact wound healing. Additionally, since different growth factors exert their effects at distinct stages of the healing process, employing the conventional ‘one-pot’ method to examine the influence of multiple growth factors leads to inaccuracies.

##### 5.4.2.3 Lysozyme & mucopolysaccharide

Lysozyme and mucopolysaccharides, such as hyaluronic acid (HA), present in saliva create a moist environment and provide antibacterial protection to the wound. Previous studies have reported high secretion of HA and lysozyme in the oral cavity. A recent study suggests that while their content differs, they exhibit similar variation trends between the oral mucosa and skin on the back (Kong et al., 2019). Furthermore, a gene expression profile analysis was conducted to compare the gene expression differences between the oral mucosa and skin on the back of rats. Surprisingly, the gene expression of hyaluronic acid and lysozyme in the oral mucosa exhibited downregulation without any significant differences, contrary to the previously reported high levels of these substances in the oral cavity. This finding suggests that the oral mucosal units are not responsible for the production and secretion of hyaluronic acid and lysozyme. In the long-term natural development process, mucosal units exhibited a weaker ability to produce and secrete these substances compared to the skin units.

##### 5.4.2.4 Salivary ions

Many studies have focused on the function of ions on wound healing, including calcium, zinc, iron, copper, etc (Dalisson and Barralet, 2019). Inorganic ions or molecules play crucial roles as catalysts and structural components in certain proteins, enzymes, and transcription factors. They can modulate the expression level and activity of these biomolecules, sometimes by inducing conformational changes. Saliva contains a rich variety of ions, among which specific nitrates have been attracting the attention of researchers (Eisenbrand et al., 2022).

Many oral bacteria have been found to express the gene for nitrate reductase and are capable of reducing nitrates to nitrites (NO_2_^−^) in vitro cultures. This nitrate reduction activity can inhibit pathogenic bacteria in the oral cavity and play a role in regulating the oral microbiome (Chang et al., 2019; Wu et al., 2019). Besides, when the oxygen utilization rate decreases and nitric oxide synthase activity is reduced, NO_2_^−^ can be converted back to nitric oxide (NO). This leads to the exertion of various physiological effects of NO, including promoting angiogenesis, clearing reactive oxygen species, and facilitating wound healing (Wu et al., 2021).

While environmental factors have been demonstrated to influence mucosal healing, they do not seem to be the primary determinant. Certain growth factors, including EGF, TGF-α, and VEGF, present in saliva, exhibit lower concentrations within the oral cavity compared to skin tissue. Smaller mucosal injuries displayed nearly identical healing rates regardless of the presence or absence of saliva, suggesting that saliva primarily contributes to the healing of larger mucosal injuries. Moreover, as previously mentioned, when skin fibroblasts are transplanted into the oral cavity, they retain the regenerative properties observed in skin tissue. This evidence suggests that the inherent responsiveness of skin and mucosal cells to regeneration plays a more significant role in wound healing.

### 5.5 Angiogenesis differences

Neovascularization plays a vital role in tissue repair and wound healing. However, excessive and robust pro-angiogenic stimuli can surpass the physiological healing demands, resulting in excessive healing characterized by substantial ECM deposition, excessive angiogenesis, and altered cellular behavior (Korntner et al., 2019). Elevated level of VEGF have been demonstrated to induce scar formation by disrupting ECM homeostasis, leading to impaired degradation and excessive deposition (Wu et al., 2004). The oral mucosa demonstrates lower levels of VEGF and reduced vascularization behavior compared to skin regeneration following injury (Szpaderska et al., 2005). Subsequent studies revealed a greater extent of hypoxia in skin wounds compared to oral mucosa, resulting in elevated levels of HIF-1α. Furthermore, hyperbaric oxygen treatment (HBOT) failed to mitigate this difference, indicating that distinct responses to hypoxia may account for the divergent wound healing characteristics observed in the skin and oral mucosa (Chen et al., 2012). Conversely, in non-injured tissues, the oral mucosa exhibited higher level of vascular endothelial markers, such as CD31 and α-SMA. Furthermore, the blood vessels in the oral mucosa are in closer proximity to the basal lamina (Chen et al., 2012). Therefore, it can be speculated that blood vessels in the oral cavity facilitate the rapid delivery of growth factors and oxygen, leading to accelerated re-epithelialization and reduced hypoxia. Consequently, the oral mucosa provides more favorable natural conditions for healing compared to the skin due to its more abundant vascular structure, resulting in a relatively lower requirement for angiogenesis during tissue repair. However, the precise mechanism underlying the reduced angiogenesis has not been fully elucidated, and it may involve endogenous anti-angiogenic factors, keratinocyte regulation, and other potential mechanisms.

### 5.6 Difference in transcription regulators

By introducing wounds on both the oral mucosa and the skin and performing biopsies sequentially at progressive time points to compare oral and skin wound healing *in vivo*, molecular and histological aspects of wound healing in paired samples of oral mucosa and skin from healthy individuals were characterized (Iglesias-Bartolome et al., 2018). Changes in gene expression profiles suggested that the homeostatic oral mucosa is endowed with a transcriptional network for wound repair in epithelial cells even before injury occurs. The data suggest that the most important processes driving acute wound repair are driven by keratinocytes. This transcriptional network is determined by the regulation of different expression patterns by a set of transcriptional regulators in oral *versus* skin keratinocytes, suggesting that transcriptional pathways established during development are responsible for different wound healing abilities of cells in different tissues. That indicates transcriptional regulators are at the center of oral wound healing and hold the key to triggering the molecular mechanisms that speed wound repair.

This study also examined the role of two transcription factors, SOX2 and PITX1, in controlling the networks associated with wound closure in oral keratinocytes (Iglesias-Bartolome et al., 2018). SOX and PITX transcription factors played crucial roles in development, influencing cell fate determination, axis formation, and pattern formation (Gage et al., 1999; Julian et al., 2017). SOX2 served as a broad marker of pluripotency and was associated with various adult stem/progenitor cell types, indicating its potential role in activating common target genes and pathways that were crucial for maintaining their self-renewal and differentiation abilities. Additionally, SOX2 was often genetically amplified in cutaneous tissues (Arnold et al., 2011; Boumahdi et al., 2014). Moreover, the research findings demonstrated that SOX2 promoted the expansion of K5+ basal/stem cell compartments in mouse skin, suggesting a connection between SOX2’s wound healing capacity and its role in regulating stem cells. ITX1 was also found to regulate a distinct set of activities associated with the level of structural proteins, such as keratins, late cornified envelope (LCE) proteins, and small proline-rich region (SPRR) proteins. Based on these findings, it was plausible to hypothesize that re-programming skin keratinocytes using SOX2 and PITX1 could enable them to acquire the properties of oral keratinocytes, leading to accelerated scar-free wound healing. However, additional research was required to elucidate the precise mechanisms by which several transcriptional regulators were activated and controlled during wound healing.

## 6 Related factors in ulcer healing

**FIGURE 4:**
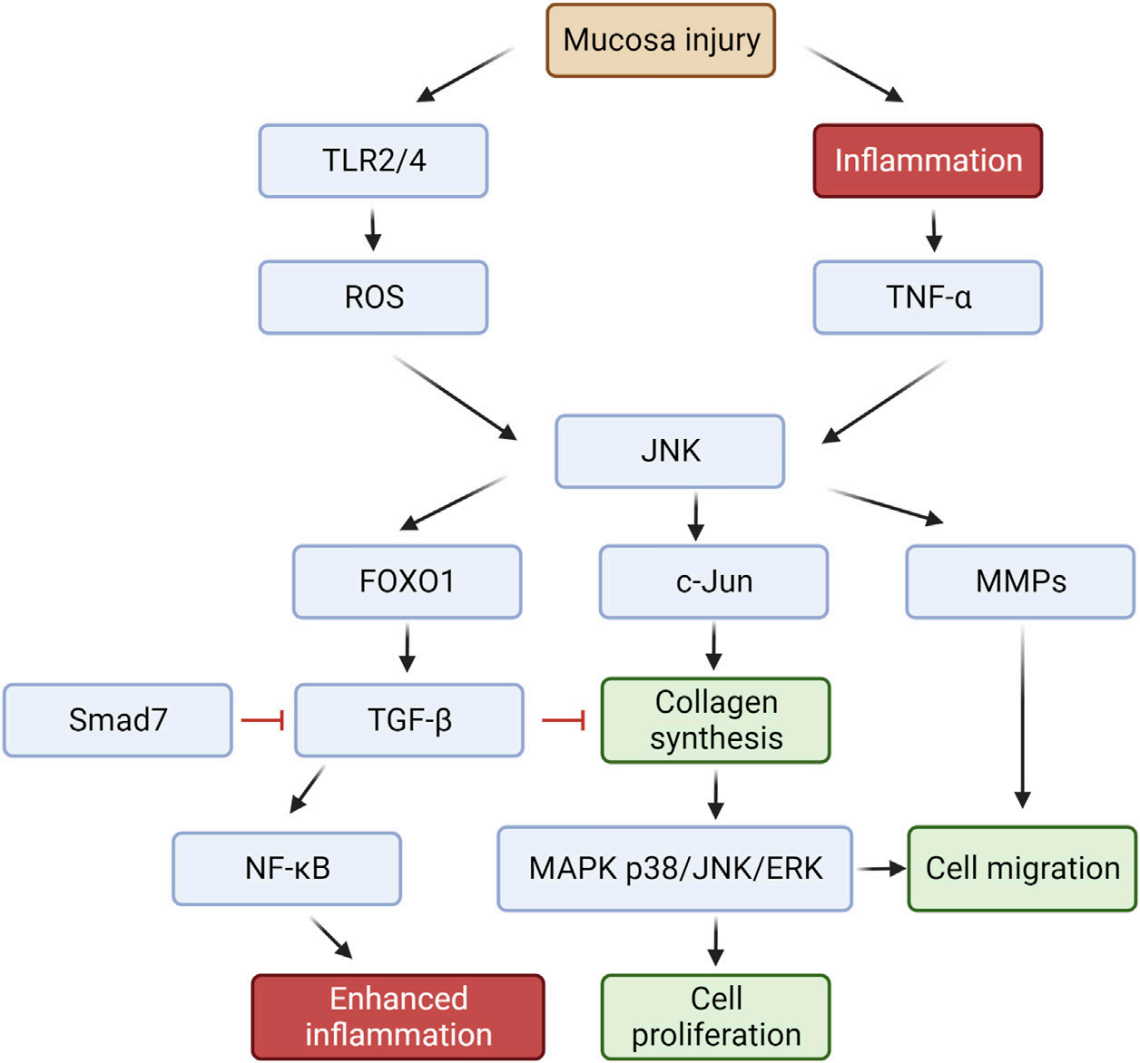
Signaling pathways and potential functions of oral ulcer-related factors.

The pathophysiology of mouth ulcers involves multiple potential causes and signaling pathways. The formation and healing of oral ulcers are influenced by various factors, including external signaling cues, immunological factors, cell surface receptor molecules, intracellular signaling, and other related variables and pathways. Various factors contribute to the formation and healing of oral ulcers (Figure 4).

### 6.1 TGF-β

TGF signaling governs various biological functions, such as proliferation, differentiation, tissue homeostasis, and regeneration. It regulates multiple processes involved in wound healing, including epidermal and dermal cell proliferation, epithelial cell migration, ECM synthesis, and immune response, during different phases of the healing process. In mammals, TGF-β is classified into three subtypes: TGF-β1, TGF-β2, and TGF-β3. Among them, TGF-β1, synthesized by platelets, lymphocytes, endothelial cells, fibroblasts, and other cell types, plays a crucial role in regulating ulcer progression.

TGF-β suppresses the growth of various cell types, such as epithelial, endothelial, hematopoietic, and glial cells, while promoting the growth of specific mesenchymal cells (e.g., skin fibroblasts) and other select cell types (Huang and Huang, 2005). Additionally, TGF-β induces chemotaxis in fibroblasts, neutrophils, and macrophages during trauma, alters the cytokine production profile of macrophages, and prompts fibroblasts to secrete extracellular matrix proteins like collagen and fibronectin. Furthermore, TGF-β1 promotes collagen synthesis and the deposition of other matrix components while suppressing the secretion of matrix MMPs and inducing the expression of proteinase inhibitors, thus impeding collagen degradation (Radwan-Oczko et al., 2008). Younai et al. observed a phenomenon where the introduction of a TGF-β1 antibody into the scar effectively suppressed the response of scar fibroblasts to TGF-β1, leading to reduced collagen synthesis and consequently inhibiting scar formation (Younai et al., 1994).

TGF-β is also crucial in regulating the inflammatory response. TGF-β stimulates the transforming growth factor TGF-β activated kinase 1 (TAK1) kinase and its binding protein, leading to the activation of the mitogen-activated protein kinase (MAPK) family (extracellular signal-regulated kinase (ERK), c-Jun N-terminal kinase (JNK), and p38) and NF-κB gene expression. This activation regulates cell survival and proliferation, while also inducing immune cell activation to produce pro- or anti-inflammatory cytokines and chemokines (Groeger and Meyle, 2019). For instance, activation of NF-κB signaling pathways by TGF-β1 induces significant inflammation in the oral mucosa. Moreover, excessive expression of *TGF-β1* in keratin-forming cells leads to impaired wound healing, which subsequently delays ulcer healing in cases of elevated TGF-β levels in oral mucositis (Wang et al., 2006).

### 6.2 Smad 7 (small mothers against decapentaplegic 7)

**FIGURE 5:**
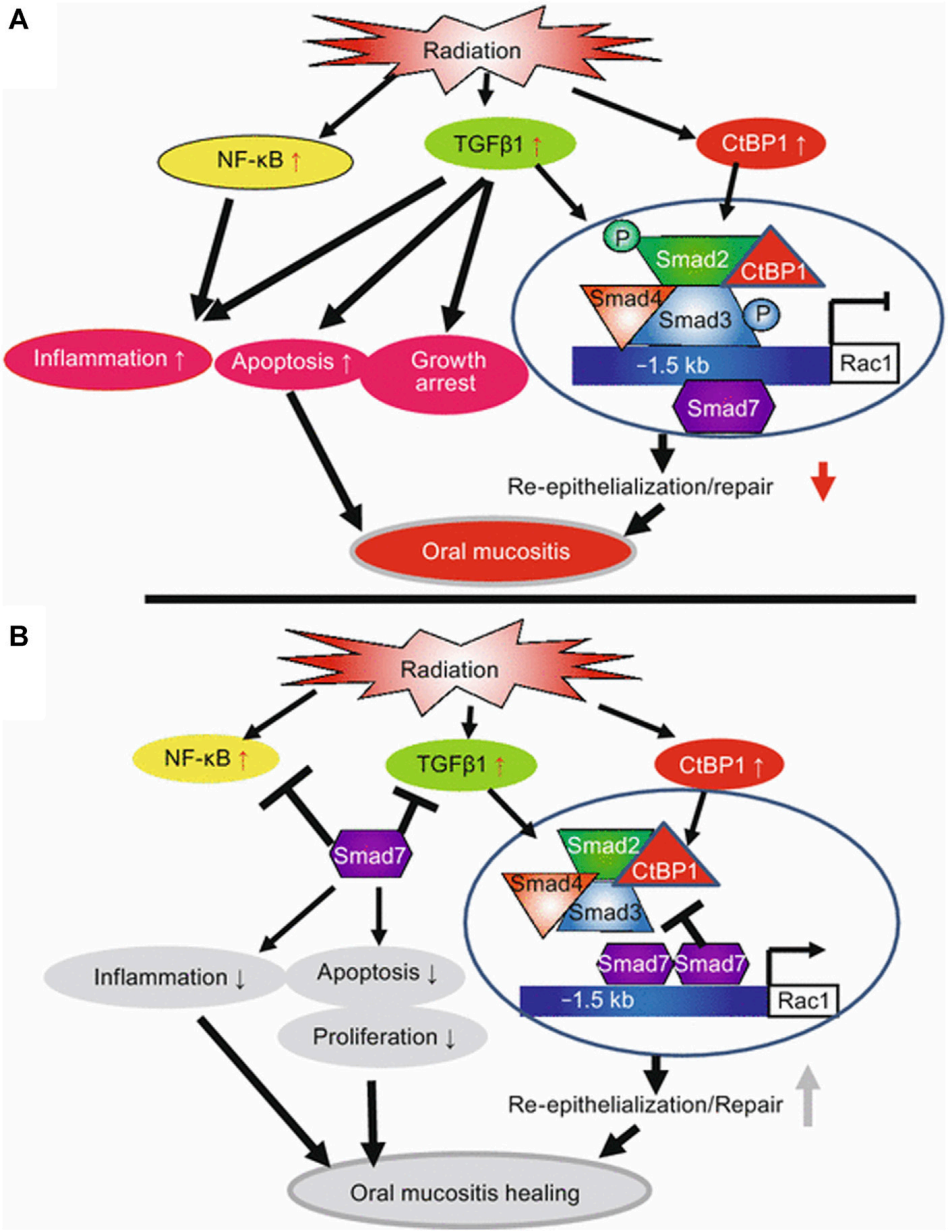
Summary of potential mechanisms of Smad7-mediated protection and healing of oral mucositis (from Han et al., Nature Medicine, 2013). **(A)** Radiation activates NF-κB, increases TGFβ1 and CtBP1. NF-κB and TGFβ1 induce inflammation. TGFβ1 induces apoptosis, growth arrest, and activates Smads which recruit CtBP1 to the Rac1 promoter to repress Rac1 transcription, leading to blunted re-epithelialization. **(B)** Smad7 blocks NF-κB and TGFβ1-induced inflammation and blocks TGFβ1-induced apoptosis and growth arrest. Smad7 activates *Rac1* by either preventing TGFβ1-mediated Smad phosphorylation or competing with signaling Smads/CtBP1 transcriptional repression complex in the *Rac1* promoter. Increased *Rac1* induced by Smad7 contributes to keratinocyte migration during re-epithelialization. Copyright (2015) Springer.

Normal keratinocytes exhibit minimal level of Smad7, but it is commonly overexpressed in pathological conditions. *Smad7* overexpression promotes epithelial cell proliferation, reduces apoptosis, and can counteract TGF-β-induced growth inhibition and apoptosis, thereby facilitating wound healing (Bian et al., 2015). Elevated levels of Smad7 upregulate the expression of *IκBα*, an NF-κB inhibitor, thereby directly counteracting the principal NF-κB inflammatory pathway (Figure 5). Additionally, Smad7 disrupts the formation of the TRAF2/TAK1/TAB2/TAB3 protein complex (TNF-receptor associated factor 2/TGF-β activated kinase 1/TAK-binding protein2/3), a crucial step in the activation of the inflammatory cascade (Hong et al., 2007). Therefore, Smad7 acts as an antagonist to TGF-β and NF-κB activity, making it one of the most effective anti-inflammatory molecules in stratified epithelial tissues. Moreover, Smad7 interacts with other pathways involved in promoting cell migration necessary for wound healing. For example, it enhances the formation of the p38 MAPK complex and the recruitment of adenomatous polyposis coli (APC) protein to microtubules.

### 6.3 FOXO1 (forkhead box protein O1)

The transcription factor FOXO1 regulates various cell types including keratinocytes, neutrophils, macrophages, dendritic cells, regulatory T cells (Tregs), and B lymphocytes, working collaboratively to maintain and repair the epithelial barrier. Research evidence demonstrates the essential role of FOXO1 in mucosal repair, particularly in re-epithelialization. Following injury, TGF-β is promptly released, and FOXO1 is crucial for wound healing as it upregulates *TGF-β* expression in keratinocytes, facilitating epithelial migration to cover the wound surface (Ponugoti et al., 2013). Additionally, FOXO1 stimulates the expression of other relevant components involved in epithelial cell migration, such as Integrins-β3, Integrins-β6, MMP-3, and MMP-9. Moreover, FOXO1 facilitates epithelial regeneration by inducing the expression of glutathione peroxidase (*GSH-Px*), which exhibits antioxidant activity, and cellular hemoglobin. This induction aids DNA repair and enhances resistance to oxidative stress. Furthermore, FOXO1 serves as a significant transcription factor in epithelial cells and contributes to connective tissue healing by generating growth factors, including VEGF, TGF-β, and connective tissue growth factor (CTGF).

### 6.4 TNF-α

TNF-α is traditionally recognized as a pleiotropic pro-inflammatory cytokine produced by various cells. However, in the context of skin wound healing studies, TNF-α has been found to contribute to skin fibroblast activation following trauma, despite its primary association with inflammatory cells and processes (Nikoloudaki et al., 2020). Studies have demonstrated that TNF-α activates JNK signaling in dermal fibroblasts. This was supported by evidence showing that fibroblasts lacking the *JNK* gene exhibited diminished migratory capacity, which was further confirmed by inhibiting JNK signaling in human dermal fibroblasts (Javelaud et al., 2003). This indicates that the presence of TNF-α in the microenvironment can facilitate the recruitment of fibroblasts into the granulation tissue through JNK activation during the process of wound healing.

Moreover, during inflammation, the pro-inflammatory cytokine TNF-α has the ability to enhance the level of MMPs and expedite tissue degradation. MMPs play a crucial role in both tissue remodeling and degradation of the extracellular matrix. While an excessive presence of MMPs may impede wound healing, unbound MMPs can facilitate basal membrane remodeling, cell migration, and regeneration of epidermal cells (Laura et al., 2012). Consequently, TNF-α has the potential to enhance fibroblast migration and stimulate epithelial cell regeneration through the activation of JNK signaling and upregulation of MMP expression, thereby facilitating the healing of mucosal tissues.

### 6.5 JNK

JNK, a transcription factor belonging to the MAPK family, plays a crucial role in the intracellular response of eukaryotic cells to various stimuli from the external cellular microenvironment. TGF-β-mediated collagen production in cells is linked to the activation of downstream C-Jun phosphorylation subsequent to JNK activation. Preventing c-Jun phosphorylation would impede TGF-β-induced collagen production (Lagares et al., 2010). However, Dolivo et al. presented contrasting findings by demonstrating that the inhibition of JNK triggers the activation of fibroblasts in human dermal fibroblasts (Dolivo et al., 2019). In summary, the mechanism underlying JNK activation in fibroblasts remains elusive.

After skin or mucosal injury, the release of TNF-α and other pro-inflammatory factors triggers JNK activation, thereby facilitating collagen synthesis and cell migration in epithelial and fibroblastic cells. Consequently, this process expedites tissue re-epithelialization and repair. However, a study has shown that inhibiting JNK can mitigate the disruption of the epithelial barrier and suppress the release of inflammatory factors (Guo et al., 2014). Furthermore, TNF-α stimulates T cells to produce multiple inflammatory factors while also facilitating the synthesis of collagen and MMPs through the JNK/c-Jun pathway, thereby contributing to tissue repair. Therefore, the signaling pathways interact with one another, necessitating a delicate balance between promoting the inflammatory response and facilitating tissue repair in order to promote ulcer healing.

## 7 Local treatment agents

Symptomatic therapy forms the cornerstone of local treatment for mouth ulcers. Local therapy aims to achieve analgesia, alleviate pain, and facilitate tissue regeneration.

### 7.1 Analgesics

While analgesics do not directly promote healing, they still play a significant role in managing symptoms. Patients with major aphthous ulcers often experience severe pain and encounter difficulties with eating, as these ulcers typically require a month or longer to heal. Lidocaine and dacronin hydrochloride are commonly used analgesics in clinical practice. Moreover, the over-the-counter medication Zilactin (Zila Inc, Phoenix, AZ) incorporates a hydroxypropyl cellulose coating that exhibits both mucosal adhesion properties and prolonged analgesic effects (Rodu and Russell, 1988). However, a drawback of its application is the transient burning sensation it causes on the mucous membranes.

### 7.2 Corticosteroids

Corticosteroids are widely recognized as the primary treatment modality for mouth ulcers. Corticosteroids can typically resolve most mouth ulcers within a week, and effectively manage severe outbreaks, but have limited efficacy in preventing recurrence. *In vitro* studies have shown that dexamethasone or triamcinolone acetonide alone can promote ulcer healing (Ryu et al., 2020; An et al., 2022). Moreover, a clinical trial has demonstrated that the use of triamcinolone in conjunction with fluocinonide gel accelerates ulcer healing and extends the inter-recurrence period (Pimlott and Walker, 1983). However, it is crucial to note that prolonged use of corticosteroids at high systemic doses should be avoided to mitigate significant adverse effects.

### 7.3 Immunomodulators

Thalidomide acts as a glutamate derivative with analgesic, immunomodulatory, and anti-inflammatory properties. It is believed that thalidomide lowers TNF levels, thereby mitigating the immune system’s attack on healthy mucous membranes (Pedersen et al., 1992). In a clinical trial conducted by Revuz et al., it was found that patients who received thalidomide experienced significant improvement, although it did not prevent relapse (Revuz et al., 1990). In addition to its well-known teratogenic side effects, thalidomide is associated with various other adverse effects, such as headache, constipation, dry mouth, and drowsiness. Consequently, thalidomide’s reputation hinders its popularity.

### 7.4 Nonsteroidal anti-inflammatory drugs (NSAIDs)

In the treatment of ulcerative colitis using salazosulfapyridine (SASP), mesalazine (5-aminosalicylic acid) serves as the active component. Collier et al. demonstrated that mesalazine can effectively alleviate discomfort, pain, and promote healing in patients with oral ulcers, with no significant occurrence of adverse effects (Collier et al., 1992). The mechanisms of action are proposed to involve the reduction of prostacyclin synthesis, suppression of oxygen metabolite generation by polymorphonuclear cells, and inhibition of leukotriene release from mucosal membranes.

### 7.5 Regenerative therapy

Mesenchymal stem cells (MSCs) have been employed in cellular therapy for the treatment of cutaneous and mucosal wounds. This treatment has demonstrated effectiveness through mechanisms such as cell differentiation and the release of paracrine factors (Komori et al., 2005; Yaojiong et al., 2010). Lee et al. recently introduced adipose-derived MSC sheets as a potential alternative therapy for accelerating the healing process of oral mucosal ulcers, with the ability to potentially replace current treatment modalities. The potential mechanism of action may lie in the ability of MSCs to differentiate into various cell types and exert paracrine effects, including anti-apoptotic, pro-angiogenic, and stem cell activation functions at the site of injury. Moreover, MSCs may inhibit TGF-β-induced apoptosis and suppress the activation of the NF-κB signaling pathway at the wound sites (Lee D. Y. et al., 2017). Although this method is user-friendly and painless, it is susceptible to technical costs, conditions, and ethical considerations.

### 7.6 Traditional Chinese medicines

Traditional Chinese medicines have shown great potential in the treatment of oral ulcers (Lee Y.-C. et al., 2017). For example, Astragalus membranaceus has been proven to regulate various signaling pathways through multiple molecule targets to treat oral ulcer via network pharmacology and molecular docking-based investigation (Zhong et al., 2023). Many traditional Chinese medicines have also demonstrated excellent pain control effects, offering significant prospects for the treatment of oral ulcers (Yuan et al., 2015; Guo et al., 2021). However, the mechanism of action of these traditional Chinese medicines in the treatment of oral ulcers is not yet fully understood. In the future, more basic and clinical research studies should be conducted to further advance the application of traditional Chinese medicine in the field of oral ulcers.

## 8 Mucoadhesive mechanisms and polymers

Due to the complex oral environment, drugs need to bind with carriers to achieve sustained-release effects. Nowadays, there are various types of carriers based on polymeric materials, such as films, tablets, powders, patches, pastes, ointments, hydrogels, and so forth. The building blocks of these polymeric materials are polymers capable of interacting with mucosal surfaces. The oral adhesive substance serves a dual purpose: firstly, as a physical barrier to prevent additional injury to the ulcer, and secondly, as a platform for sustained medication release. Therefore, careful material selection is crucial. The term ‘bioadhesion’ was initially introduced by Longer and Robinson in 1986, and it refers to the attachment of a synthetic or natural macromolecule to the mucus and/or an epithelial surface (Longer and Robinson, 1986). More specifically, the general definitions of “bioadhesive” and “mucoadhesive” remain unchanged, referring to the interaction between a polymer and a biological surface, and the adherence between a polymeric material and mucosal tissue, respectively.

**FIGURE 6:**
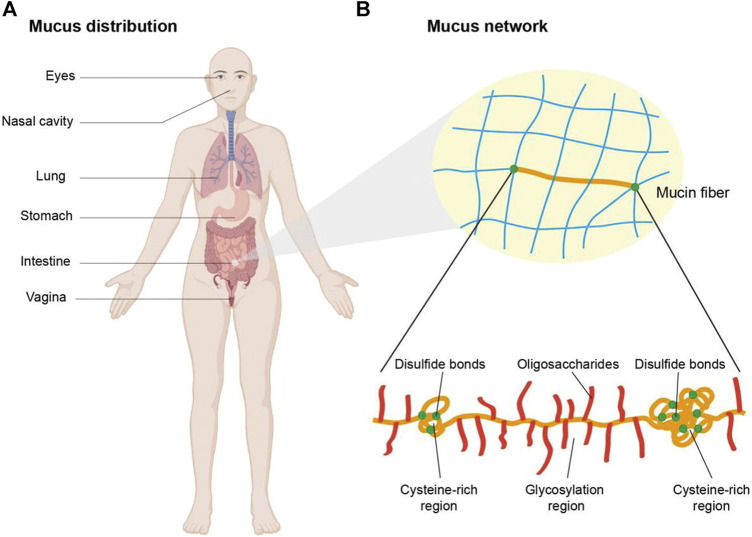
Schematic illustration of the mucus distribution and structure. **(A)** Mucus is distributed in eyes, nasal cavity, lung, stomach, intestine, etc. **(B)** The network of mucus is composed of mucin fibers which contain glycosylation regions with negative charge and non-glycosylated hydrophobic regions rich in cysteine. Copyright (2021) Elsevier.

The mucosal surface is enveloped by a hydrated gel-like layer known as mucus, predominantly comprised of mucin (Figure 6). Mucin consists of peptide backbones and oligosaccharide side chains, with the backbone featuring a repeated sequence domain referred to as PTS, representing proline, threonine, and serine residues. The O-linked oligosaccharide side chains typically terminate in sialic acid or sulfonic acid residues (Gandhi and Robinson, 1994). Due to the deprotonation of carboxyl groups, mucins in saliva generally carry a negative charge. Two prominent theories, namely, adsorption and diffusion theories, are recognized to have significant implications in bioadhesion (Peppas and Buri, 1985; Gu et al., 1988; Ahuja et al., 2008).

According to absorption theory, primary chemical bonds are formed through non-covalent interactions, including electrostatic forces, van der Waals’ forces, hydrogen bonds, and hydrophobic interactions, when polymers come into initial contact with mucus or biological interfaces. Subsequently, as the polymer expands in the hydrated environment, it penetrates the mucus, leading to the formation of secondary chemical bonds facilitated by various polymers that will be discussed later (Huang et al., 2000). The flexibility of polymer chains and the characteristics of ionizable groups significantly impact the adhesion force. The diffusion theory posits that the adhesion force arises from the entanglement between the glycoprotein chains of mucin and the polymer chain. Upon initial contact, the bioadhesive polymer chains diffuse into the mucus network, creating an entangled structure between the two polymers. The flexibility of the polymer chain, diffusion coefficient, chemical structure, and degree of exposure may serve as crucial factors influencing the strength of interaction (Smart, 2005).

**FIGURE 7:**
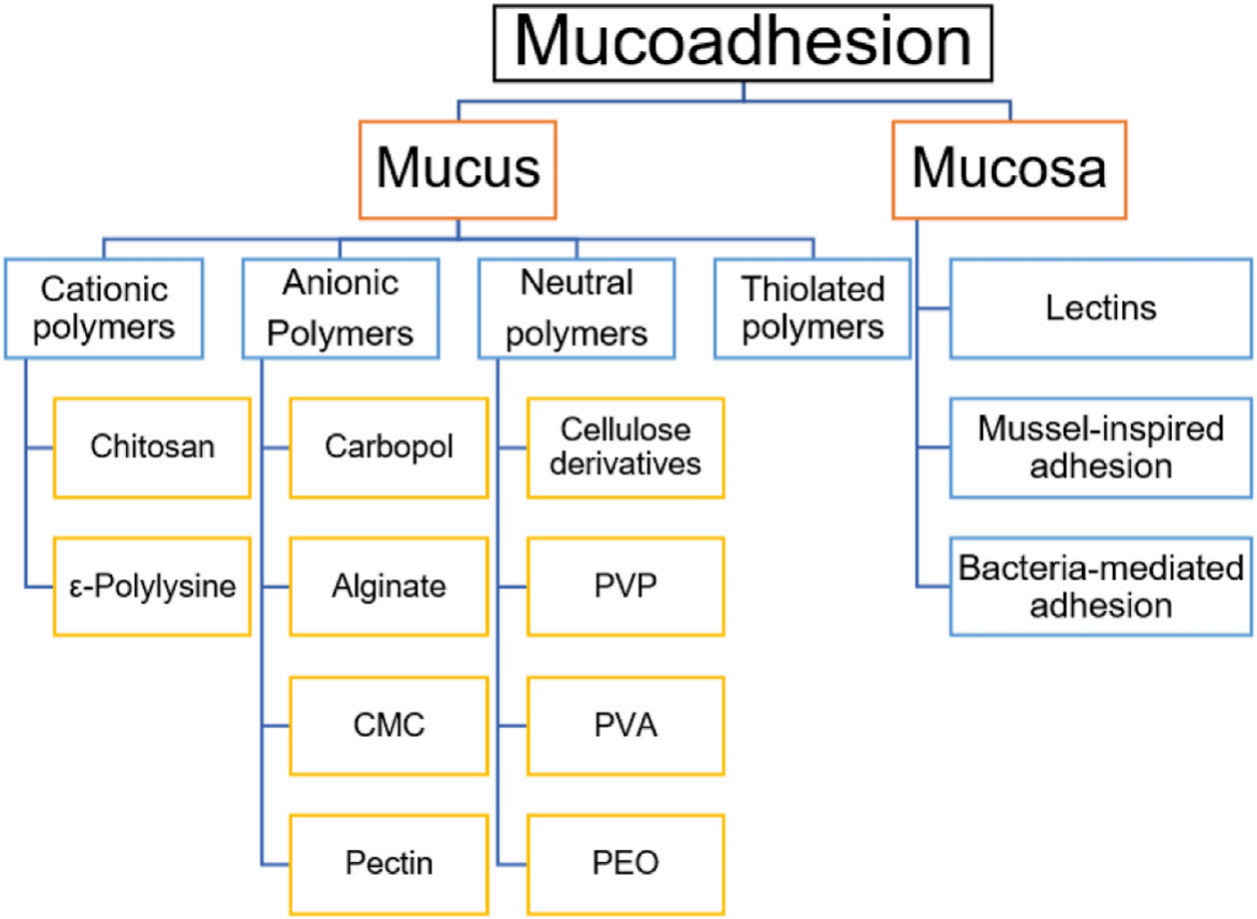
Classification of mucoadhesive mechanisms and polymers.

Various approaches can be employed to achieve the desired adhesion effect in complex environments. The following strategies can be considered, and combining them can result in stronger adhesion forces (Figure 7).

### 8.1 Adhesion to the mucus layer

As mentioned earlier, the mucus layer is characterized by acidity and primarily comprises mucin chains abundant in cysteine residues. Subsequently, we will delve into various adhesion substrates based on distinct polymer characteristics.

#### 8.1.1 Cationic polymers

Cationic polymers possess the inherent ability to adhere to negatively charged mucus through electrostatic interactions. An exemplary cationic polymer is chitosan (CS), which is derived from chitin via partial deacetylation in an alkaline environment. Chitosan is composed of N-acetyl-glucosamine and d-glucosamine units. With a pKa value of approximately 6.5, chitosan is insoluble in water but readily dissolves in acidic solutions. This solubility arises from the protonation of its primary amines, resulting in the formation of positively charged polyelectrolytes or polycations, which confer mucoadhesive properties to chitosan (Dash et al., 2011).

The solubility of chitosan improves with an increase in the degree of deacetylation. Moreover, the increased number of free amine groups can be employed to form complexes with polyanions (Mun et al., 2016). A previous study observed that chitosan microspheres exhibited higher adherence to the intestine at low crosslink densities, which aligns with the presence of a greater number of free amino groups in chitosan. Furthermore, deacetylation was found to enhance chitosan’s adhesion properties, whereas crosslinking reduced mucosal adhesion (George and Abraham, 2006). A study demonstrates that the interaction between chitosan and mucin is complex and involves various mechanisms such as hydrogen bonding and hydrophobic interactions, with electrostatic attraction playing a predominant role (Collado-González et al., 2019). Lehr et al. also suggest that higher molecular weight contributes to mucoadhesion. Furthermore, repeated usage does not result in a significant decline in adhesiveness (Lehr et al., 1992).

Chitosan is widely regarded as a highly versatile carrier derived from nature due to its biocompatible, biodegradable, non-toxic, mucoadhesive, penetration enhancement, and non-immunogenic properties. It plays a significant role in tissue engineering and drug delivery. Noha et al. developed a buccal patch utilizing chitosan and polyvinylpyrrolidone (PVP), with a thickness of approximately 0.7 mm and a diameter of 10 mm. The patch remains on the mucosa for up to 3 h and exhibits a release profile of up to 67.63% of miconazole nitrate within 5 h (Nafee et al., 2003). However, its limited solubility in aqueous solutions restricts its application. Specifically, at physiological pH, chitosan loses its cationic properties, thereby diminishing its ability to enhance drug permeability and absorption (Pawar and Jaganathan, 2014). By leveraging the presence of hydroxyl and amine groups, chitosan can be modified to overcome the solubility challenge. One such modification involves grafting a glycol branch, resulting in glycol chitosan (GC). This derivative exhibited water solubility at both neutral and acidic pH levels, with the glycol branch enhancing solubility beyond that of natural chitosan and providing spatial stability. Moreover, GC enhances the mucoadhesive properties attributed to its higher zeta potential, which preserves its cationic character and improves interaction with the mucus (Palazzo et al., 2017).

ε-Polylysine (EPL) is another significant cationic polymer known for its favorable biocompatibility and strong adhesion to various biological surfaces (Ciucurel and Sefton, 2011). Its cationic properties enable interactions with negatively charged cell membrane surfaces, making it valuable as an adhesive material for cell culture and a carrier in oral drug delivery. In a study by Xu et al., a mucoadhesive hydrogel was developed using heparin-modified poloxamer as a substrate and EPL as a functional excipient. This hydrogel aimed to facilitate the delivery of growth factors, expediting the healing process of endometrial injury (Xu et al., 2017). The rheological properties and mucoadhesive behavior of the hydrogel can be easily adjusted by varying the EPL content. The hydrogel exhibits a maximum adhesion force of 3.18N, which is ten times higher than that of the EPL-free hydrogel. Additionally, the inclusion of EPL significantly enhances the release of growth factors compared to the heparin-modified poloxamer hydrogel.

#### 8.1.2 Anionic polymers

Initially, the utilization of anionic polymers may appear counterintuitive due to the negative charge of the mucus layer. Nevertheless, numerous anionic polymers have demonstrated robust mucoadhesive properties. Generally, the carboxyl and sulfate groups present in anionic polymers acquire a negative charge when the solution’s pH surpasses the polymers’ pKa. This phenomenon is commonly attributed to the entanglement between the polymer chains and mucin fibers, although emerging evidence suggests that hydrogen bonds also play a significant role (Fefelova et al., 2007). Notably, polyacrylates (Carbopol), alginate, and carboxymethylcellulose are extensively employed anionic polymers known for their high negative charge density and excellent cushioning capacity. However, a significant drawback of anionic polymers is their incompatibility with multivalent cations such as Mg^2+^, Cu^2+^, and Zn^2+^.

Carbopol, a high molecular weight polyacrylic acid (PAA) polymer, is crosslinked with allyl sucrose or allyl pentaerythritol to act as a tissue-adhesive and thickening agent with rapid, high, and stable swelling properties (Bonacucina et al., 2004). However, its mechanical strength may be compromised due to its elevated swelling rate and solubility. To address this, poloxamer is added to decrease the water solubility of Carbopol while maintaining adhesion through hydrogen bonding interactions. In a study by Chun et al., a polymeric oral patch composed of Carbopol, poloxamer, and hydroxypropyl methylcellulose (HPMC) was developed. This formulation effectively reduced the swelling and dissolution rates of the polymer films, enabling adhesion on the mucosa for over 8 h (Chun et al., 2003).

Alginate is a natural anionic mucoadhesive polysaccharide derived from brown seaweed and has been used for wound healing (Zhu et al., 2021; Yan et al., 2022; Dou et al., 2023; Wang et al., 2023). It can form gels by crosslinking β-d-mannuronate (M) and α-l-guluronate (G) blocks, which have different molecular weights and ratios. The mechanical properties of the alginate gel can be controlled by adjusting the ratio of these blocks. The properties of the G block, such as its sequence, molecular weight, and length, also affect the properties of the gel. Alginate’s physical properties play a role in drug release, stability, and cell entrapment within the gel (Nayak et al., 2013). In studies, the size of alginate beads ranged from 0.92 ± 0.05 to 1.30 ± 0.14 mm, with drug encapsulation efficiency ranging from 71.63% ± 2.32% to 95.08% ± 3.73%. The release rate of drugs from the beads over 10 h varied from approximately 69.78% ± 2.43% to 95.70% ± 4.26%. After an 8-h immersion in a pH 1.2 hydrochloric acid solution, around 50%–70% of the beads remained adhered to the intestinal mucosa.

CMC is an anionic mucoadhesive polysaccharide used as a cost-effective thickening agent and solubilizing substrate in the pharmaceutical industry. It blends well with other polymers due to its high solubility (Baldwin et al., 2011). In a buccal film for ibuprofen delivery, a blend of CMC and PVP showed good results *in vitro* and *in vivo*. The film achieved a maximum adhesion time of 5.5 h on mucosa and 5 h on the gingival (Perioli et al., 2004). It also exhibited good hydration performance and released ibuprofen into saliva. Another study developed a buccal film blend using HPMC as the substrate and incorporating carbopol-934P, Eudragit RL-100, and SCMC individually. The film with HPMC and SCMC demonstrated appropriate mucosa residence time and drug release performance (Semalty et al., 2008).

Pectin is a mucoadhesive anionic polymer. A study compared the mucosal adhesion behavior of different anionic polymers, including pectin, sodium alginate, and SCMC (Ali and Bakalis, 2011). Various parameters such as concentration, molecular weight, ionic strength, and contact time affect the adhesion effect. Among these anionic polymers, pectin has the lowest viscosity, maximum pulling force, and maximum detachment force, while sodium alginate demonstrates the highest mucoadhesion effect. Another study investigated the interaction between mucin and six types of pectin from different manufacturers, which had varying degrees of methoxylation and amidation substitution (Hagesaether and Sande, 2007).

#### 8.1.3 Neutral polymers

Nonionic polymers, like anionic polymers, adhere to the fluid environment through chain entanglement and hydrogen bonding. However, the non-covalent interactions are not as robust as those observed in cationic and anionic polymers.

Cellulose derivatives like CMC, HEC, HPC, HPMC, and MHEC are commonly used synthetic polymers. Most of these derivatives are non-ionic, except for CMC, which is an anionic polymer. Among them, HPMC is widely utilized and consists of O-methylated and O-(2-hydroxypropylated) cellulose building blocks. The presence of methoxyl and hydroxypropyl groups alters the physical properties of HPMC oral films. Hydroxypropyl promotes hydration, while methoxy is more hydrophobic, leading to the formation of a gel-like structure (Joshi, 2011). Increasing the methoxy content enhances properties such as stiffness, resistance, and ductility. HPMC types K and E are commonly used in oral films, where type K is suitable for drug release control and type E serves as a film-forming agent (Panraksa et al., 2020).

Recent developments involve double-layered films using HPMC, PVA, and chitosan loaded with ornidazole and dexamethasone sodium phosphate for treating oral ulcers. These films exhibit favorable swelling properties, achieve high drug release rates, and reduce inflammation without mucosal irritation (Zhang et al., 2019). Adjusting the concentration, type, and copolymerization of HPMC can yield desirable properties for polymer films, offering versatility (Dahiya et al., 2009). HPMC films have been found to possess greater elasticity, toughness, and adhesiveness compared to CMC-based films (Peh and Wong, 1999).

HPC is a cellulose derivative obtained through the partial substitution of hydroxyl groups in cellulose with hydroxypropyl moieties. The molecular weight of HPC ranges from approximately 50,000 to 1,250,000. Due to its favorable swelling properties, HPC films exhibit adjustable adhesion, drug-carrying capacity, and reasonable clarity (Dahiya et al., 2009). Moreover, HPC has a notable characteristic of high solubility in various solvents, significantly broadening the range of drugs that can be loaded.

In recent decades, several synthetic non-ionic polymers, such as PVA, PVP, polyethylene oxide (PEO), and methacrylate polymers, have been investigated as film-forming agents (Langoth et al., 2003). Varied concentrations of these polymers can influence mucoadhesion, physical properties, and drug release characteristics. However, the potential pro-inflammatory properties of synthetic polymers restrict their use in the biomedical field.

#### 8.1.4 Thiolated polymers

The carboxyl and amino terminal domains of mucin contain numerous cysteine residues, which can potentially form disulfide bonds with thiolated mucus adhesion polymers (Bernkop-Schnürch et al., 1999). Thiolated polymers, such as PAA, PAAm, PVA, CMC, pectin, chondroitin, and hyaluronic acid, exhibit mucoadhesive properties, enhanced tensile strength, cohesiveness, swelling behavior, and water uptake characteristics (Bernkop-Schnürch et al., 2004). However, current thiol groups, including thioglycolic acid, L-cysteine, and imine sulfhydryl compounds, have shown unsatisfactory pro-inflammatory potential *in vivo*.

One notable example is sulfhydrylated chitosan, which includes four distinctive compounds: chitosan-thioglycolic acid (CS-TGA), chitosan-4-thiobutyl-amidine (CS-TBA), chitosan-cysteine (CS-Cys), and chitosan-thioethylamidine (CS-TEA) (George and Abraham, 2006). Thiolated chitosan modifies the primary amino or amidine group at the 2-position of the glucosamine subunit using coupling agents with thiol functionality. Sulfhydryl-modified chitosan forms stable adhesion with mucin through disulfide bonds, surpassing the electrostatic effect of chitosan and mucus layers. However, CS-TGA is prone to inadvertent oxidation, reducing polymer adhesion. Hence, performing the reaction at pH < 5 or in an inert environment is recommended. Similarly, CS-TBA, modified with 2-iminothiane, exhibits enhanced mucoadhesive properties, with adhesion force increasing as pH decreases (Bernkop-Schnürch et al., 2004). Pre-activated sulfur polymers have been developed to mitigate the oxidation of sulfhydryl groups (Laffleur et al., 2013). These polymers, attached to the chitosan backbone, pre-activate thiol groups via disulfide bonds in the presence of 6,6-dithionicotinamide. Compared to unmodified chitosan, CS-TBA shows significantly longer adhesion time and extended drug release time. Overall, thiolated polymers, particularly sulfhydrylated chitosan and its derivatives, offer promising mucoadhesive properties and can enhance drug release times.

### 8.2 Adhere to the mucosa

#### 8.2.1 Lectins

During the 1980s, adhesion polymers became widely used in drug delivery, leading to the development of many commercial products. However, a significant limitation of these adhesives is their lack of substrate specificity when interacting with the mucus layer. They adhere to both mucus and dislodged mucus in the same way, without distinguishing between different surfaces. As a result, non-specific mucoadhesives can adhere to unintended surfaces, hindering their adhesion to the intended mucosal tissue interfaces (Lehr, 2000). This issue is particularly prominent in gastrointestinal and oral applications, where premature inactivation can occur, limiting the durability of mucus adhesives to the rapid turnover of mucus. Achieving effective adhesion to the mucosa is challenging due to the complex motor and secretory activities in this organ.

Thus, an exceedingly attractive approach for targeted administration involves the advancement of bioadhesive polymers that can selectively interact with specific targets, such as receptors on cell membranes in particular tissues. Cell adhesion, which bypasses reliance on mucus turnover, could be achieved if it were possible to discover novel molecules that directly bind to the cell membrane, rather than adhering to the “unreliable” mucus gel layer, via specific receptor-ligand interactions.

A new generation of adhesion polymers has been developed, including lectins. Lectins are proteins or glycoproteins that can selectively recognize sugar molecules and bind to glycosylated membrane components like glycoproteins and glycocalyx. Unlike mucus-sticking polymers that adhere to the mucus gel network, lectins and other adhesion molecules specifically recognize receptor-like components on the cellular membrane. This means that they directly bind to epithelial cells themselves, known as cell adhesion (Phillips et al., 1982). Additionally, these site-specific interactions with receptors can trigger intercellular communication, leading to the internalization of drugs or carrier systems (via endocytosis through cell adhesion) into lysosomes and potentially other cellular compartments, such as the nucleus.

Lectin-like compounds, called “lectinomimetics,” have been developed to understand the binding and internalization mechanisms of epithelial cells. Computer modeling reveals that most remaining glycoproteins do not participate in lectin-sugar interactions. Smaller lectinomimetics with reduced immunogenicity and toxicity have been pursued to achieve cellular attachment and internalization (Lehr, 2000). These interactions can be incorporated into target-specific bioadhesive polymers for specific cell attachment and inhibition of non-specific interactions with mucins. Future research on second-generation adhesive polymer lectins and lectinomimetics should explore their applications in precise targeting and cellular internalization.

#### 8.2.2 Mussel-inspired bioadhesion

Marine mussels are being explored as a potential source of effective tissue adhesives in the biomedical field due to their remarkable adhesion abilities underwater. They secrete adhesion proteins that bind to surfaces in seawater, creating strong adhesive patches. Among these proteins, mussel foot protein (mfp)-1 to mfp-6 are the main contributors to the robust and moist adhesion between the plaque and substrate. These proteins contain diverse residues like 3,4-dihydroxy-*L*-phenylalanine (DOPA), tyrosine, phenylalanine, and various charged groups. In particular, mfp-5 has the highest concentration of DOPA, accounting for 30% (Zhang et al., 2020). DOPA is considered the primary adhesive primer due to its exceptional wet adhesion properties. The catechol group in DOPA can form both covalent and non-covalent interactions, making it highly functional during the crosslinking process (Hu et al., 2021). It can create non-covalent compounds like hydrogen bonds and metal ligands. Under oxidizing or basic conditions, the catechol moiety undergoes oxidation, forming o-quinone. This oxidized form can establish covalent bonds with nucleophilic substances such as thiols and amines on tissue surfaces through Michael addition or Schiff base reactions. O-quinone can also form bi-dopa crosslinks through phenol radical coupling.

**FIGURE 8:**
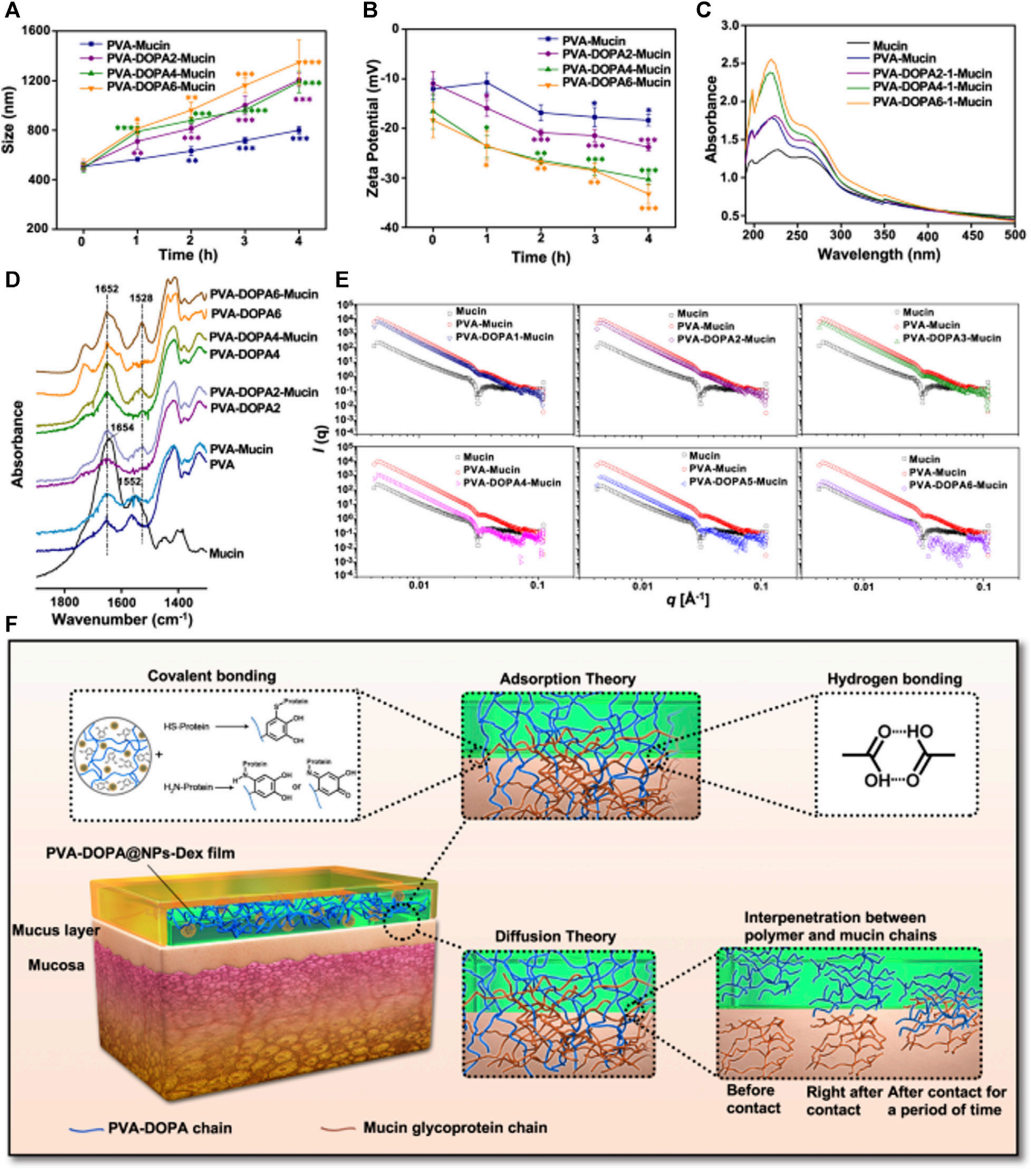
**(A)** Variation in the particle size of different PVA-DOPA-Mucin mixtures as a function of time. PVA: poly (vinyl alcohol), DOPA: 3,4-dihydroxy-D-phenylalanine. n = 3 independent samples per group; *p = 0.025; **p < 0.01; ***p < 0.001 vs. value at 0 h **(B)** Variation in the zeta potential of different PVA-DOPA-Mucin mixtures as a function of time. n = 3 independent samples per group; *p < 0.05; **p < 0.01; ***p < 0.001 vs. value at 0 h **(C)** UV-vis absorbance spectra of different PVA-DOPA-Mucin mixtures. **(D)** FTIR spectra of different PVA-DOPA before and after mixed with mucin. **(E)** SAXS spectra of different PVA-DOPA before and after mixed with mucin. **(F)** Schematic overview of the interactions between the PVA-DOPA film and mucus. NPs: nanoparticles, Dex: dexamethasone. All data are Mean ± S.D. Statistics was calculated by one-way ANOVA followed by Tukey’s post-test. Copyright (2021) Nature.

A recent study explored the mucoadhesive properties of a novel oral patch inspired by mussels (Hu et al., 2021). The study introduced a tunable film called PVA-DOPA, composed of a mixture of the mussel adhesion protein DOPA and the mucosal adhesion polymer PVA. The film demonstrated strong adherence to moist oral tissues for at least 8 h in rat models, with a maximum adhesion force of 38.72 ± 10.94 kPa. The film’s rapid erosion limited the data collection to 8 h. Additionally, polydopamine (PDA)-coated PLGA nanoparticles were developed and found to efficiently traverse the mucus layer and epithelial cells. This facilitated targeted release of dexamethasone for treating localized oral ulcers. The dense polydopamine coating reduced entanglement with mucin fibrils and minimized interaction with negatively charged structural domains of mucin. The negatively charged phenolic groups of polydopamine interacted with positively charged choline groups on the lipid membrane, while the positively charged amino groups of polydopamine bound to negatively charged phosphate groups on the epithelial cell surface. This improved the absorption of PLGA-PDA nanoparticles by cells (Figure 8).

Moreover, another study also paid attention on the adhesive properties of a mussel-inspired oral patch (An et al., 2022). The hydrogel exhibited high toughness and strong wet adhesion force. The catechol structure of dopamine reacted with amine and sulfhydryl groups on the tissue surface, resulting in strong adhesion. The hydrogel matrix dissipation was attributed to physical cross-linking of gelatin, chemical cross-linking of gelatin with polydopamine through Michael addition and Schiff base reaction, and molecular chain binding induced by nano-clay.

In a recent article, a coenzyme-based polymer binary elastomer adhesive patch was developed for the treatment of oral ulcer (Cui et al., 2023). α-Lipoic acid (LA) is a coenzyme in mitochondria related to energy metabolism. This study created a PolyLA-Na/PolyLA binary synergistic system to establish robust wet tissue adhesion via multiple electrostatic interactions and hydrogen bonds between carboxyl groups and the amino groups on the mucosa.

#### 8.2.3 Bacteria-mediated adhesion

Initially, microorganisms were thought to cause illnesses, but scientific research has shifted our perception of microbes. Most bacteria, except for a few pathogenic strains, inhabit different parts of the body and serve specific purposes. Bacteria employ various mechanisms to maintain physiological balance in the human body. Researchers have proposed that studying bacterial behavior, in line with the principles of bionics, can provide valuable insights into human beings.

The adhesive properties of bacterial cells, particularly their fimbriae, have recently been investigated. Fimbriae are specialized appendages that enable bacteria to adhere to other cells or surfaces (Scheller and Cotter, 2015).

Pathogenic bacteria use fimbriae to attach to specific receptors and exert their pathogenic effects (Savage, 1977). Similar to lectins, this bacterial-mediated adhesion helps retain drugs within the mucus layer and facilitates interaction with cellular receptors. Some bacteria have shown strong adhesive characteristics. For example, *Escherichia coli* specifically adheres to the lymphoid follicular epithelium of the ileal Peyer’s patch in rabbits (Inman and Cantey, 1983). Certain staphylococci preferentially adhere to the mucus layer rather than the cell membrane (Sanford et al., 1989).

In one study, poly (acrylic acid) polymer modified with the K99 antigen, a fimbrial protein from *E. coli*, significantly slowed down the migration of equine erythrocytes through the K99-poly (acrylic acid) gel compared to a PAA gel. This suggests a strong affinity of the K99 protein for erythrocyte receptors. (Bernkop-Schnürch et al., 1995).

In addition to adherence, some bacteria demonstrate receptor-mediated endocytosis, known as bioinvasion. Cross-linking invasin, a surface protein from *Yersinia pestis*, to model particles increased their uptake by Madin-Darby Canine Kidney (MDCK) cells compared to non-cross-linked particles. Researchers have patented the use of bacteria as carriers to efficiently internalize drug carriers into host cells through interactions with β1 chain integrin cell receptors. This bioinvasive drug delivery system shows promise for targeted drug administration (Xu et al., 2023). With advances in biotechnology, the development of a unique drug delivery system that modulates microbial cytokines and enables cytosolic entry into cells through bacterial adhesion factors holds potential. This technology brings us closer to achieving targeted oral drug administration.

## 9 Conclusion and outlook

In this review, we conclude the structure, characteristics, and potential therapies for oral ulceration. Notably, the distinctive healing properties of the oral mucosa and the utilization of oral adhesive materials are emphasized. Drawing upon the bionic theory, we believe that imitating the rapid scar-free healing mechanism observed in mouth ulceration can lead to the development of novel therapies for enhancing skin healing. Moreover, by reviewing a range of adhesive polymers and diverse adhesion processes, we aim to inspire the development of multi-component, robust, and highly adhesive drug-loading platforms.

Furthermore, the highly vascularized and immune-competent buccal mucosa presents a practical and appealing alternative route for administering potent medications. Oral administration offers benefits such as enhanced medication utilization, improved patient compliance, and ease of administering acute doses during clinical crises. Before application, extensive experiments are required to determine the therapeutic targets, and large-scale clinical trials are needed to ensure treatment efficacy. Consequently, this innovative medication delivery system holds promise for treating a range of disorders, including mouth ulcers, thereby creating new avenues for therapy. In conclusion, our endeavor has provided novel insights into the invention of innovative therapeutic approaches for oral ulceration. We aspire that 1 day, this global ailment will receive efficacious and timely treatment.
